# Cleaning Effectiveness and Postoperative Pain Associated With Rotary Instrumentation in Primary Teeth: An Umbrella Review of Systematic Evidence

**DOI:** 10.7759/cureus.87369

**Published:** 2025-07-06

**Authors:** Kavitha Swaminathan, Sushmita Shan, Esther Kirubakaran, Vaishnavi Padmanabhan, Ramanathan Ravi, Selvakumar Haridoss

**Affiliations:** 1 Pediatric and Preventive Dentistry, Sri Ramachandra Institute of Higher Education and Research, Chennai, IND; 2 Conservative Dentistry and Endodontics, Manipal University College Malaysia, Melaka, MYS

**Keywords:** amstar 2, cleaning effectiveness, meta-analysis, pediatric dentistry, postoperative pain, pulpectomy, rotary instrumentation, systematic review, umbrella review

## Abstract

Rotary instrumentation in primary teeth pulpectomy is gaining popularity due to its potential to improve procedural efficiency and clinical outcomes. While existing systematic reviews and meta-analyses (SRMAs) have explored rotary techniques, comparative evaluations of specific clinical endpoints, particularly cleaning effectiveness and postoperative pain, remain fragmented. This umbrella review synthesised evidence from SRMAs assessing rotary versus manual instrumentation in primary teeth, focusing on these two critical outcomes. A comprehensive literature search was conducted across six databases and grey literature until May 2025. Two eligible SRMAs were included: one addressed both cleaning effectiveness and postoperative pain, while the other focused solely on cleaning outcomes. Owing to clinical and statistical heterogeneity, meta-analysis was not feasible. Methodological quality appraisal using AMSTAR-2 rated one review as high confidence and the other as low confidence. According to the GRADE assessment, the certainty of evidence was low for cleaning effectiveness and moderate for postoperative pain, primarily due to inconsistency and imprecision. The findings suggest that rotary instrumentation may offer advantages in obturation quality and postoperative comfort in primary teeth. However, the limited number of high-confidence SRMAs and heterogeneity in outcome reporting underscore the need for standardised research protocols in pediatric endodontics.

## Introduction and background

Primary teeth endodontics aims to preserve the health, function, and integrity of primary teeth until their natural exfoliation [1]. This is especially critical as primary teeth play a vital role in maintaining arch integrity, phonation, mastication, and guidance of permanent successors [2]. Traditional hand instrumentation in primary teeth pulpectomy is often time-consuming, operator-dependent, and uncomfortable for young patients, leading to procedural fatigue and potential behavioral challenges during treatment [3]. With the introduction of nickel-titanium (NiTi) rotary instruments in pediatric dentistry, significant improvements have been noted in shaping ability, reduction of chairside time, and procedural predictability, thereby improving patient compliance and clinical outcomes [4].

Several systematic reviews and meta-analyses have compared rotary versus manual instrumentation in primary teeth, focusing on various parameters such as instrumentation time, obturation quality, and apical debris extrusion [5]. Chugh et al. highlighted the improved obturation quality with rotary systems [6], whereas Gala et al. emphasised reduced postoperative pain and procedural efficiency [7]. However, many of these studies are limited by methodological inconsistencies, in vitro designs, or broad outcome scopes, often lacking a comprehensive synthesis of clinically relevant endpoints such as postoperative pain and cleaning efficacy. Two important outcome measures in pulpectomy are cleaning effectiveness, the ability to remove necrotic tissue and debris from the root canal and postoperative pain, a key indicator of patient comfort and procedural success. Cleaning effectiveness is often inferred from obturation quality, which reflects how completely and homogeneously the canal is filled, and may also be influenced by apical debris extrusion, the unintended pushing of debris beyond the root apex that can provoke inflammation or pain. A growing body of literature indicates that rotary instrumentation may provide superior debridement and fewer procedural errors, yet the quantitative evidence supporting these benefits in primary teeth, particularly concerning cleaning effectiveness and patient-reported pain, remains fragmented and inconclusive.

In contrast to the umbrella analysis by Patnana et al. [8], which provided a broad overview of clinical outcomes associated with rotary and manual instrumentation in pediatric endodontics, the present review specifically addresses two core outcomes: cleaning effectiveness and postoperative pain in primary teeth. Furthermore, our study incorporates a more rigorous methodological appraisal using both AMSTAR-2 and GRADE frameworks, alongside a quantitative assessment of review overlap via the corrected covered area (CCA). These additions enhance the reliability and specificity of our findings, offering clearer clinical implications for primary tooth pulpectomy.

## Review

Materials and methods

This umbrella review was conducted following the Preferred Reporting Items for Systematic Reviews and Meta-Analyses (PRISMA) guidelines. The protocol was registered with the International Prospective Register of Systematic Reviews (PROSPERO; ID: CRD42024590194).

Literature Search

A comprehensive search was performed across six major databases: PubMed, Scopus, Cochrane Library, Web of Science, Google Scholar, and OpenGrey. The final search was executed on May 26, 2025. The search strategy used a combination of keywords and Medical Subject Headings (MeSH) terms including “pulpectomy,” “primary teeth,” “rotary instrumentation,” “manual instrumentation,” “cleaning effectiveness,” and “postoperative pain.” Boolean operators (AND/OR) were used to combine terms, and search filters were applied for language (English), study type (systematic reviews and meta-analyses), and human studies.

Eligibility Criteria

Studies were selected based on the PICOS framework. The population included children undergoing pulpectomy in primary teeth. The intervention was rotary instrumentation, compared against manual or hand instrumentation. The primary outcomes assessed were cleaning effectiveness and postoperative pain. Only systematic reviews and meta-analyses (SRMAs) that included randomised controlled trials (RCTs) conducted on primary teeth were eligible for inclusion. In vitro studies, narrative reviews, and studies involving permanent teeth were excluded.

Study Selection and Data Extraction

All retrieved records were imported into Rayyan for screening. Two independent reviewers performed title and abstract screening, followed by full-text assessment. Discrepancies were resolved through discussion or consultation with a third reviewer. Data were extracted into a standardised Excel sheet, including author details, publication year, sample characteristics, outcome measures, and statistical findings. Cleaning effectiveness was operationalised through obturation quality (e.g., optimal fill, absence of voids), while postoperative pain was measured at defined intervals (6 hours, 12 hours, 24 hours).

Methodological Quality Appraisal

The quality of the included systematic reviews was appraised using a measurement tool to assess systematic reviews (AMSTAR-2 ), which is a validated instrument for evaluating systematic reviews that include randomised or non-randomised studies of healthcare interventions [9]. AMSTAR-2 includes 16 domains, of which 7 are considered critical (e.g., protocol registration, adequacy of the literature search, assessment of risk of bias, and methods for meta-analysis). Each item was evaluated independently by two reviewers, with disagreements resolved through discussion. Reviews were classified as high, moderate, low, or critically low confidence based on the presence of critical and non-critical weaknesses, as per AMSTAR-2 guidance.

Certainty of Evidence Assessment

The certainty of evidence for each outcome was assessed using the grading of recommendations assessment, development and evaluation (GRADE) framework [10]. This approach evaluates five domains-risk of bias, inconsistency, indirectness, imprecision, and publication bias-for each outcome of interest. Based on this appraisal, the quality of evidence was categorised as high, moderate, low, or very low. Two reviewers independently performed the GRADE assessment, with consensus reached through discussion.

Data Synthesis and Analysis

A quantitative synthesis (meta-analysis) was planned for outcomes reported across multiple SRMAs. However, due to considerable heterogeneity in definitions, measurement units, and comparators, a meta-analysis was not performed for cleaning effectiveness and postoperative pain.

Results

Study Selection

**Figure 1:**
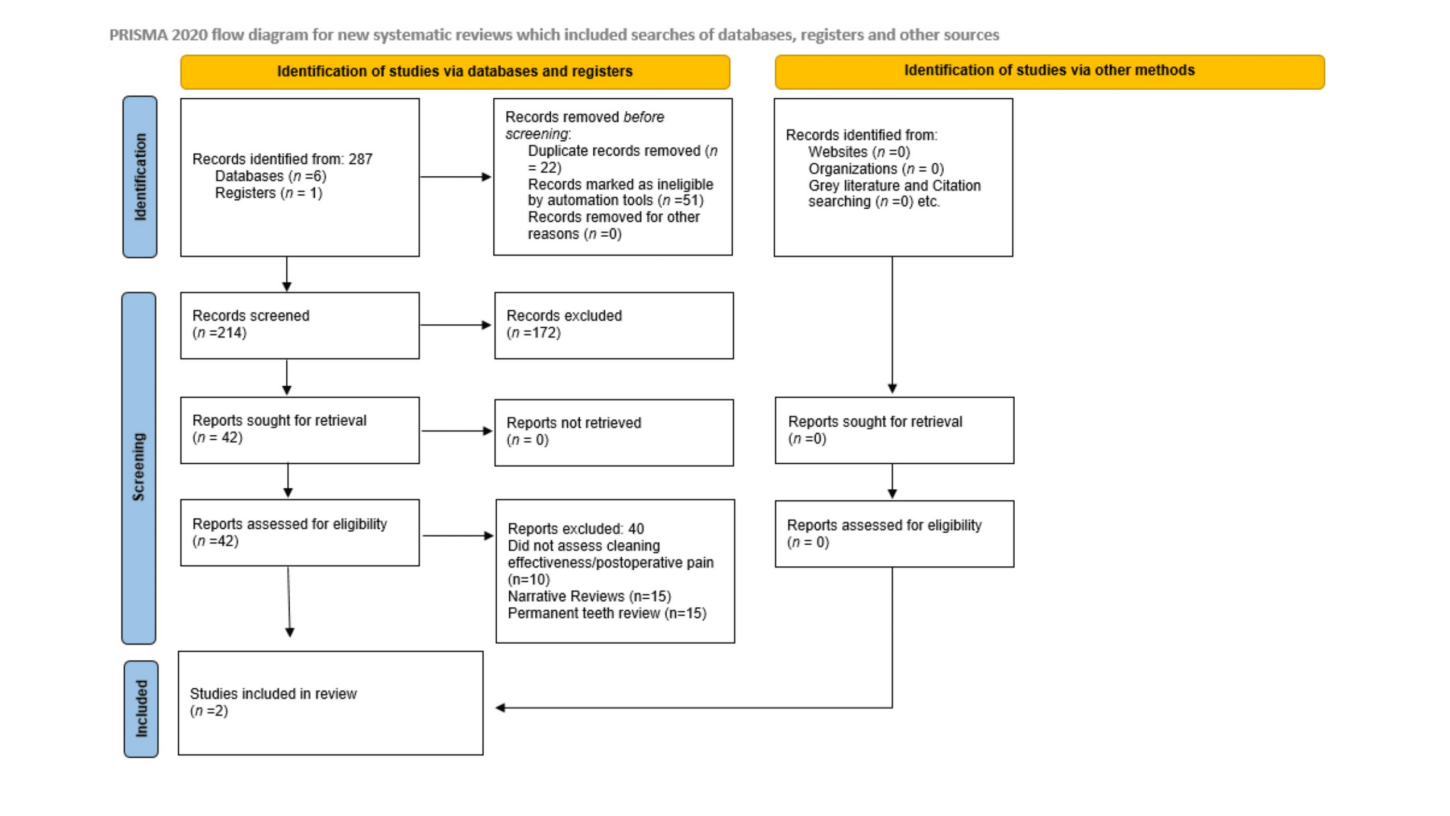
PRISMA 2020 flow diagram for study selection. The PRISMA flow diagram outlines the study selection process for this umbrella review. A total of 287 records were identified through database and register searches. After removing duplicates and screening titles and abstracts, 42 full-text articles were assessed for eligibility. Of these, 40 were excluded. Ultimately, two systematic reviews and meta-analyses met the inclusion criteria and were included in the final synthesis.

The initial search yielded 287 records from six databases and one register. After removing 22 duplicates and 51 records excluded by automation tools, 214 records were screened. Following title and abstract review, 42 full-text articles were assessed for eligibility. Ultimately, two systematic reviews and meta-analyses (SRMAs) met the inclusion criteria for this umbrella review. A PRISMA 2020 flowchart summarises the selection process (Figure 1).

Study Characteristics

The two included SRMAs, Khadmi et al. [11] and Faghihian et al. [12], evaluated outcomes related to rotary versus manual instrumentation in pulpectomy of primary teeth. While all included meta-analyses of randomised controlled trials (RCTs), each focused on different outcomes. Khadmi et al. assessed both cleaning effectiveness and postoperative pain, and Faghihian et al. exclusively analysed cleaning effectiveness. The rotary systems assessed in the included studies varied (e.g., ProTaper, Kedo-S), and outcome measures were defined in terms of obturation quality and postoperative pain evaluated at specific intervals (6 hours, 24 hours, and 48 hours). Table 1 summarises the key characteristics of the included SRMAs, including study design, population, rotary systems used, and outcomes assessed.

**Table 1:** Key characteristics of included systematic reviews and meta-analyses. This table summarises the essential characteristics of the two included systematic reviews and meta-analyses comparing rotary and manual instrumentation in primary teeth. Details presented include author and publication year, study design, number of primary studies included, population characteristics, type of rotary systems evaluated, outcomes assessed (cleaning effectiveness and postoperative pain), and methods of synthesis. Both studies focused on pulpectomy procedures in primary teeth using various outcome definitions and measurement intervals. ZOE: zinc oxide eugenol; Metapex: iodoform-calcium hydroxide paste; Ca(OH)₂: calcium hydroxide.

Author(s)	Year	Article title	Journal	Study type	Sample size	Age range	Rotary system used	Comparator (if any)	Outcome: cleaning effectiveness (reported/not reported)	Outcome: postoperative pain (reported/not reported)	Cleaning effectiveness measure	Postoperative pain measure	Obturation materials used	Effect size (cleaning)	Effect size (pain)	Risk of bias	Comments	Full text available (yes/no)
Khadmi I et al. [11]	2025	Different outcomes of rotary and manual instrumentation in primary teeth pulpectomy: a systematic review and meta‑analysis	European Archives of Paediatric Dentistry	Systematic review and meta‑analysis	2936	3 to 12 years	WaveOne system, ProTaper Next system, Rotary Hyflex EDM® Files, K3 Ni–Ti files, ProDesign Logic file, Kedo S rotary NiTi file, Kedo S square files, RaCe rotary system, Prime Pedo TM files, Protaper Universal TM, HERO Shaper rotary files, Pedo-Flex paediatric rotary files, Kedo-SG paediatric rotary files, Pro-AF Baby Gold paediatric rotary files, S2 Protaper rotary system, Mtwo rotary system, Fanta AF™ Baby rotary files, Kedo-SG blue files, Flexicon X7 file system, Hyflex CM file system, Reciprocating files, Rotary Flex Master System, HeroShaper Rotary File, XP-endo Shaper files, Pro Taper Gold rotary files, Kedo-SG Blue rotary files, Revo-S rotary files, Wave One GOLD files (reciprocating motion), Light Speed LSX files,	Manual K files, Kedo-SH hand files, H files, Manual Ni–Ti K files	Reported	Reported	Obturation quality assessed via subgroup analysis based on unit of analysis-‘Root’ and ‘Teeth’. Also assessed void presence. Meta-analyses were performed for all.”	Binary outcome of pain absence at 6 h, 24 h, and 48 h using Wong-Baker FACES, Modified Wong-Baker, and Four-point pain intensity scales	Primarily ZincOxide Eugenol (ZOE) and Metapex; few studies used Calcium Hydroxide Ca(OH)₂ + ZOE	Root: OR=1.66 (95% CI: 1.25–2.21, p= 0.0005, I²= 49%); Teeth: OR=2.62 (95% CI: 2.00-3.44, p<0.00001, I²=0%); Voids: OR=4.29 (95% CI: 2.34-7.87, p<0.00001, I²=22%)	6 h: OR=3.37 (95% CI: 2.40–4.74, p=0.00001, I²=11%); 24h: OR=2.23 (95% CI: 1.40-3.54, p=0.0007, I²=38%); 48 h: OR=2.27 (95% CI: 1.29-3.99, p=0.004, I²=0%)	Revised Cochrane risk-of-bias tool for randomised trials (RoB 2.0)	The meta-analysis compares rotary and hand files in primary teeth pulpectomy, revealing significant differences in outcomes. A total of 47 trials were included, showing rotary instruments reduced instrumentation and obturation time. Postoperative pain incidence was significantly lower with rotary instrumentation after 6, 24, and 48 hours. No significant differences were noted in Clinical success between the two groups after 3 and 6 months.	Yes
Faghihian R et al [12]	2022	Rotary versus manual instrumentation for root canal preparation in primary teeth: a systematic review and meta‑analysis of clinical trials	Contemporary Clinical Dentistry	Systematic review and meta‑analysis of clinical trials	341	3 to 9 years	ProTaper file, K3 file, Mtwo file, hyflex‑CM Ni‑Ti file, Kedo‑S files, flex master file, light speed LSX file	Manual K files	Reported	Not Reported	Obturation quality (optimum, overfilled, underfilled); canal preparation efficacy; radiographic success"	Not Reported	ZOE in most studies; not explicitly stated for all trials	Optimum obturation: OR=3.53 (95% CI: 1.79-6.97); Overfilled: OR=0.58 (95% CI: 0.27-1.26); Underfilled: OR=0.45 (95% CI: 0.19-1.11)	Not reported	Revised Cochrane risk-of-bias tool for randomised trials (RoB 2.0)	The systematic review assessed rotary versus manual instrumentation for root canal preparation in primary teeth. Seven articles were selected for the review, indicating a focused analysis. The study found that rotary techniques reduced instrumentation time by 1.79 minutes compared to manual techniques. However, rotary files did not decrease the risk of underfilling and overfilling compared to manual files. All included articles were at high risk of bias due to a lack of allocation concealment.	Yes

Quality Assessment (AMSTAR-2)

The methodological quality of the two included systematic reviews and meta-analyses (SRMAs) was evaluated using the AMSTAR-2 tool. Khadmi et al. [11] was rated as high quality, with low risk of bias across all domains, including a comprehensive literature search, duplicate study selection, risk of bias assessment, and reporting of funding. Faghihian et al. [12] was rated as low confidence, primarily due to the absence of a registered protocol, lack of funding disclosure, and partial risk of bias integration in synthesis. The results of the AMSTAR-2 assessments were used in conjunction with GRADE to inform the strength of conclusions and evidence certainty. Table 2 summarises the AMSTAR-2 domain-level assessment for each included review.

**Table 2:** AMSTAR 2 methodological quality assessment of included systematic reviews. This table presents the domain-level evaluation of the methodological quality of the two included systematic reviews using the AMSTAR 2 (A MeaSurement Tool to Assess Systematic Reviews) instrument. Sixteen items were assessed for each review, including seven critical domains (e.g., protocol registration, risk of bias assessment, adequacy of the literature search). Responses were categorised as “yes,” “partial,” or “no.” Based on the presence of critical and non-critical weaknesses, overall confidence ratings were assigned as “high” for Khadmi et al. [11] and “low” for Faghihian et al. [12]

Domain	Khadmi et al. [11]	Faghihian et al. [12]
P1. Research questions and inclusion criteria include PICO components?	Yes	Yes
P2. Protocol established prior to conduct of the review?	No	No
P3. Justification for included study designs?	Yes	Yes
P4. Comprehensive literature search strategy?	Yes	Yes
P5. Study selection in duplicate?	Yes	Yes
P6. Data extraction in duplicate?	Yes	Yes
P7. List of excluded studies with justifications?	Yes	Yes
P8. Adequate description of included studies?	Yes	Yes
P9. Risk of bias from individual studies included in synthesis?	Yes	Yes
P10. Report on funding sources of included studies?	No	No
P11. Appropriate meta-analytical methods used?	Yes	Yes
P12. Assessment of impact of RoB on meta-analysis results?	Partial	Partial
P13. Consideration of RoB in interpretation/discussion?	Yes	Yes
P14. Explained heterogeneity"	Yes	Yes
P15. Assessment of publication bias?	No	No
P16. Conflict of interest declared?	Yes	Yes
Overall confidence rating	High	Low

Synthesis of Results

**Figure 2:**
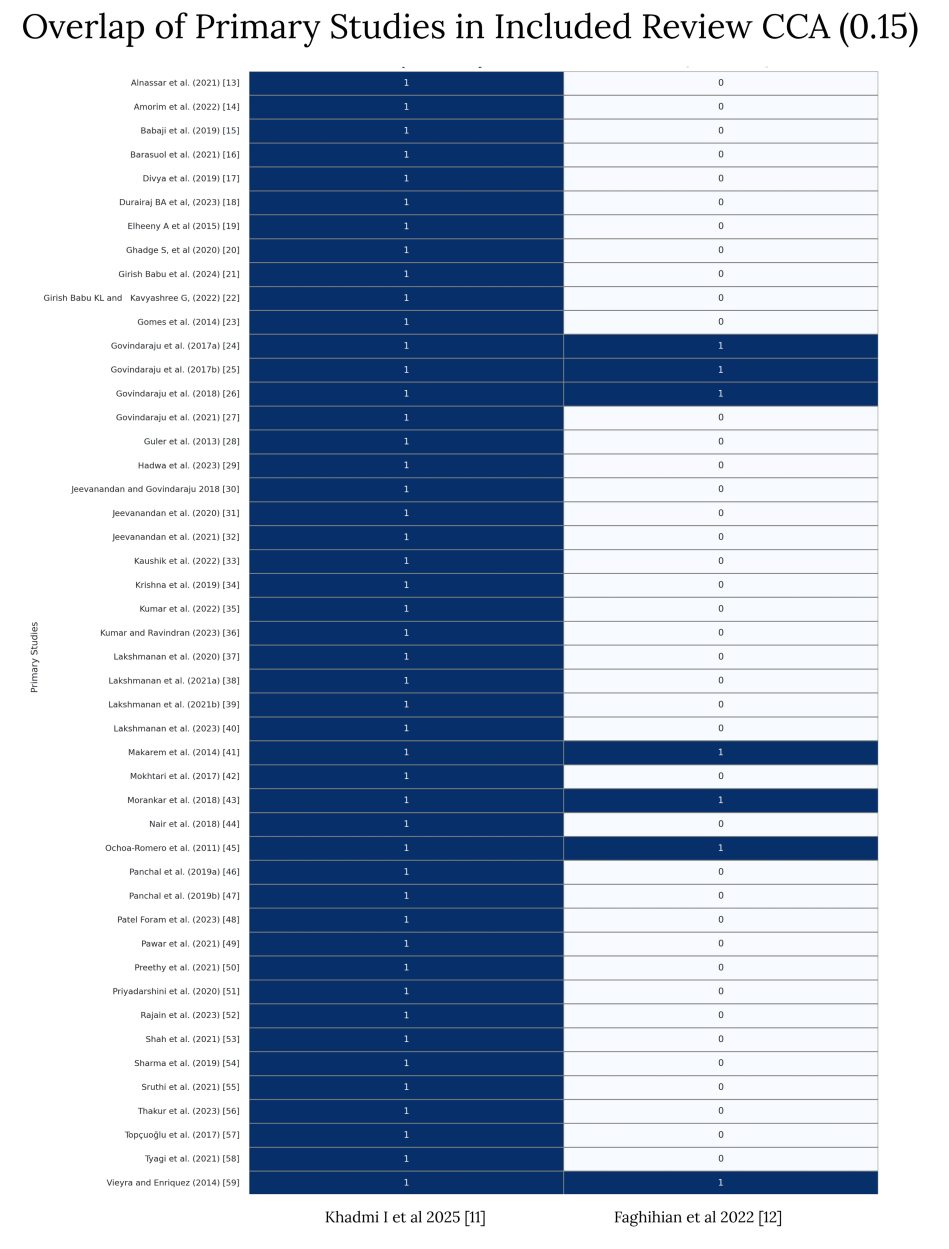
Overlap heatmap of primary studies included in systematic reviews (CCA=0.15). This heatmap displays the overlap of primary studies included in the two eligible systematic reviews: Khadmi et al. [11] and Faghihian et al. [12]. Each row represents a unique study, and each column represents a review. A value of “1” indicates inclusion of the study in the corresponding review. All seven studies included in Faghihian et al. [12] were also included in Khadmi et al. [11], resulting in a corrected covered area (CCA) of 0.15, indicating low overlap.

Due to the methodological heterogeneity, pooled analysis was not feasible. Instead, a CCA heatmap was created to visualise study overlap (Figure 2)[13-59].

The CCA between the two reviews was calculated to be 0.15, indicating low overlap in included studies. The overall certainty of evidence for cleaning effectiveness and postoperative pain was assessed using the GRADE framework. The GRADE assessment (Table 3) rated the certainty of evidence as ‘Low’ for cleaning effectiveness and ‘Moderate’ for postoperative pain due to inconsistency and imprecision in the underlying studies.

**Table 3:** Summary of findings and certainty of evidence using the GRADE framework. This table summarises the certainty of evidence for each outcome, cleaning effectiveness and postoperative pain, based on the GRADE approach. The evaluation considered five domains: risk of bias, inconsistency, indirectness, imprecision, and publication bias. Each domain was assessed to determine the overall certainty of evidence, which was categorised as “low” for cleaning effectiveness (due to inconsistency and imprecision) and “moderate” for postoperative pain. GRADE: Grading of recommendations assessment, development and evaluation.

Study	Outcome	Number of studies	Study design	Risk of bias	Inconsistency	Indirectness	Imprecision	Publication bias	Quality of evidence
Khadmi et al. [11] and Faghihian et al. [12]	Cleaning effectiveness	2	RCTs	No serious limitations	Serious	Not serious	Serious	Undetected	Low
Khadmi et al. [11]	Postoperative pain	1	RCT	No serious limitations	Not serious	Not serious	Not serious	Undetected	Moderate

Discussion

Despite notable advancements in primary teeth endodontics, the optimal instrumentation strategy for primary teeth remains a subject of clinical debate. Conventional manual files, though familiar and widely adopted, are often criticised for prolonged treatment duration and variability in obturation outcomes, particularly in younger, less cooperative patients. In contrast, rotary instrumentation offers mechanical precision and efficiency, potentially enhancing procedural consistency and reducing chairside time. While numerous systematic reviews and meta-analyses have compared these techniques broadly, few have specifically addressed cleaning effectiveness and postoperative pain, two outcomes that directly impact clinical success and patient experience. This umbrella review was undertaken to bridge this critical gap by synthesising high-level evidence focused solely on these patient-relevant endpoints, thereby refining clinical decision-making in pediatric pulpectomy protocols.

The results of this review support the potential advantages of rotary instrumentation in primary teeth. Khadmi et al. [11] and Faghihian et al. [12] reported improvements in obturation quality and reduced pain levels with rotary techniques, suggesting a more efficient and child-friendly approach. Although a consistent trend toward improved clinical parameters was observed with rotary instrumentation, the limited number of included SRMAs and outcome variability necessitate cautious interpretation. Their increasing adoption should be guided by ongoing research standardisation. Importantly, Khadmi et al. [11] provided temporal data for pain outcomes (6 hours, 12 hours, and 24 hours), adding depth to the assessment. The AMSTAR-2 and GRADE tools confirmed high methodological rigor for one review and low for the other, with evidence certainty ranging from low (cleaning effectiveness) to moderate (postoperative pain), strengthening confidence selectively.

An additional variable that may have influenced clinical outcomes across the included studies is the type of obturation material used. In the majority of the studies included in both SRMAs, Zinc Oxide Eugenol (ZOE) was the most commonly employed material. Several recent trials, particularly in Khadmi et al. [11], also utilised Metapex, an iodoform-based calcium hydroxide paste. A few studies used combinations such as Calcium Hydroxide with ZOE. The variability in obturation materials may partially account for differences in postoperative pain and obturation quality, although this was not systematically analysed in either SRMA. Future meta-analyses may benefit from stratifying outcomes based on obturation material, as some past literature suggests a possible association with apical extrusion, periapical inflammation, or radiographic success.

The clinical applicability of findings from this umbrella review is influenced not only by instrumentation type but also by operator experience and setting. In low-resource environments, access to NiTi rotary instruments, training programs, and endodontic equipment may be limited, which can affect the implementation of standardised treatment protocols. Studies have reported variations in instrumentation adoption among practitioners due to economic and logistical constraints. These disparities underscore the importance of tailoring recommendations to regional healthcare capacities and ensuring adequate clinician calibration.

Furthermore, variability in outcomes such as pain and obturation quality could be influenced by subjective measurement tools and inconsistent follow-up durations. For instance, although several studies evaluated postoperative pain, the scales and time-points used were not standardised. Bonzanini et al. (2021) rightly cautioned about the risk of canal transportation with rotary systems in curved canals, a reminder that technique sensitivity and case selection remain critical [60].

This review has several strengths, including a clearly defined protocol (PROSPERO ID: CRD42024590194), duplicate screening and quality assessment, and use of validated tools (AMSTAR-2 and GRADE). The focus on pediatric outcomes, particularly pain, adds clinical relevance often overlooked in endodontic reviews. However, our conclusions are limited by the small number of eligible SRMAs and the inability to conduct pooled effect size estimations due to heterogeneity. While findings suggest clinical benefits, certain limitations must be acknowledged

Future research should prioritise the development of core outcome sets for pediatric endodontic trials, with standardised pain metrics and consistent obturation evaluation protocols. Well-designed, multicenter randomised controlled trials are also needed to provide more homogeneous data and facilitate high-certainty meta-analyses. This umbrella review reinforces the necessity for harmonised evaluation protocols and outcome metrics in pediatric pulpectomy research, laying the groundwork for more comparable and impactful meta-analyses in the future.

## Conclusions

This umbrella review synthesised high-level evidence from two systematic reviews and meta-analyses on the effectiveness of rotary instrumentation in primary tooth pulpectomy, specifically focusing on cleaning quality and postoperative pain. While the findings favour rotary systems in terms of obturation quality and reduced postoperative discomfort, the conclusions must be interpreted cautiously due to methodological heterogeneity and variations in obturating materials across included studies. Zinc oxide-eugenol was the most commonly used obturation material, but several trials employed alternatives such as metapex and calcium hydroxide combinations, which may have influenced radiographic and clinical outcomes. The CCA value of 0.15 indicates low overlap between reviews, further highlighting the fragmented nature of the current evidence base. Future randomised controlled trials should adopt standardised pain scales such as the Wong-Baker Faces or FLACC scale, ensure consistent time-point assessments for pain outcomes, utilise core outcome sets specific to primary endodontics, and establish calibration protocols for evaluating obturation quality. Additionally, greater emphasis is needed on real-world implementation factors such as operator experience and resource availability, particularly in low-resource settings. Addressing these gaps will enhance the reliability and applicability of future research in pediatric endodontics.
